# Trastuzumab and Pertuzumab Suspected Therapy-Induced Ocular Toxicity in Patient Living with HER2-Positive Advanced Breast Cancer

**DOI:** 10.1159/cro/abzag014

**Published:** 2026-09-22

**Authors:** Lahari Ramagiri, Kai C.C. Johnson, Margaret E. Gatti-Mays, Ashley P. Davenport

**Affiliations:** a College of Medicine, The Ohio State University, Columbus, OH, USA; b Division of Medical Oncology, Department of Internal Medicine, The Ohio State University Comprehensive Cancer Center, College of Medicine, The Ohio State University Wexner Medical Center, Columbus, OH, USA

**Keywords:** Trastuzumab, Pertuzumab, Toxicity, Case report

## Abstract

**Introduction:**

Trastuzumab and pertuzumab, in combination with chemotherapy, are first-line anti-human epidermal growth factor 2 (HER2) therapies in the treatment of HER2-positive metastatic breast cancer. Although these therapies have been shown to improve the overall survival of patients, they can also be associated with adverse events; ocular toxicities have not been well-documented.

**Case Presentation:**

This report outlines the case of a patient with a spectrum of new-onset transient ocular symptoms on pertuzumab-trastuzumab combination therapy that improved with cessation of the drug and recurred with trastuzumab monotherapy rechallenge. She developed ocular symptoms 2 years into her treatment course. Her principal ophthalmologic diagnosis was dry eye syndrome of bilateral lacrimal glands and keratoconjunctivitis sicca. Ultimately, she opted for close observation in lieu of continuing therapy. Her staging scans show no evidence of disease, and she denies eye-related symptom recurrence.

**Conclusion:**

Given the limited knowledge regarding the long-term ocular adverse events of HER2-targeted therapies, medical oncologists should routinely ask patients about new-onset or worsening ophthalmic symptoms.

## Introduction

Human epidermal growth factor 2 (HER2)-positive breast cancer accounts for about 15–20% of breast cancer diagnoses. HER2 is a tyrosine kinase receptor found on the surface of breast cells, and its overexpression has been associated with aggressive tumor growth and invasion. However, anti-HER2 agents have been shown to significantly improve the overall survival and disease-free survival of HER2-positive breast cancer patients. These agents include monoclonal antibodies (e.g., trastuzumab and pertuzumab), tyrosine kinase inhibitors, and antibody-drug conjugates (e.g., trastuzumab emtansine and trastuzumab deruxtecan) [1].

According to the American Society of Clinical Oncology (ASCO) guidelines, the first-line treatment for HER2-positive metastatic breast cancer, irrespective of hormone-receptor status, is pertuzumab, trastuzumab, and a taxane (e.g., docetaxel and paclitaxel), followed by maintenance pertuzumab-trastuzumab. If a patient progresses on first-line therapy, second-line treatment is trastuzumab-deruxtecan. If a patient continues to progress, there are several third-line HER-2 targeted therapy combinations available that require shared decision-making to choose the best option based on factors like treatment schedule, toxicities, and route of delivery. For patients with HER2-positive and hormone receptor-positive metastatic breast cancer, first-line treatment with the strongest evidence is pertuzumab, trastuzumab, and a taxane (as above), with the addition of endocrine therapy when chemotherapy ends, and/or progression occurs. In select cases, physicians may recommend endocrine therapy and trastuzumab or lapatinib, or endocrine therapy alone [2, 3]. Despite the benefits of HER2 agents, adverse events can occur due to the inhibition of HER2 receptors in unaffected tissues. Ocular toxicities of HER2 agents have not been well documented. Post-marketing adverse event reports for trastuzumab include blurred vision, conjunctivitis, corneal disease, dry eye syndrome, lacrimation (irritation-related), maculopathy, and vision loss [4]. Aside from increased lacrimation in 4–5% of patients, there are no post-marketing reports of ophthalmic adverse events with pertuzumab [5].

Among the limited cases studying ocular adverse events associated with these medications is a retrospective pharmacovigilance study conducted by Chen et al. [6]. They reviewed a total of 3467 ocular adverse event reports on anti-HER2 agents in the US Food and Drug Administration (FDA) Adverse Event Reporting System (FAERS) database from 2018 to 2023. They discovered that, compared to tyrosine kinase inhibitors, antibody drugs (e.g., pertuzumab and trastuzumab) were associated with a higher risk and broader spectrum of ophthalmic pathologies, including conjunctival, corneal, optic nerve, and retinal disorders [6].

In adults, HER2 is expressed more strongly in corneal and conjunctival tissues due to the presence of epidermal growth factor receptor proteins. The pathophysiology behind anti-HER2 agents and ocular toxicities is unclear; however, one proposed hypothesis is that HER2 inhibition may cause impaired turnover of the corneal and conjunctival epithelium, leading to inflammation and ocular damage. This pathophysiology was proposed by Peeters et al. through a case report of a patient who developed corneal melting after 3 cycles of trastuzumab monotherapy [7]. Another possible mechanism was illustrated by Saleh et al., who reported a rare case of bilateral ischemic maculopathy and severe visual loss after 3 months on trastuzumab therapy that improved with cessation of the drug and recurred on rechallenge. They suggested this occurred due to trastuzumab’s ability to reduce the expression of pro-angiogenic factors, like vascular endothelial growth factor, and capillary caliber, leading to capillary leakage and macular ischemia [8]. Barmas-Alamdari et al. [9] published a case report on a patient who endorsed eyelid crusting and a sandy sensation in her eyes. She was found to have a new diagnosis of bilateral corneal ulceration with perforation of an ulcer. The patient’s symptoms improved with therapeutic keratoplasty and ocular surface therapy. The authors propose that the corneal ulceration may have occurred due to trastuzumab’s ability to inhibit epidermal growth factor receptor in the cornea. Furthermore, since the patient developed symptoms after an initial loading dose of trastuzumab, they propose that the higher dose may have been toxic to the corneal tissue. She also had diffuse metastases, including at the orbit, which may have impinged on the trigeminal nerve branches and led to a higher susceptibility to damage [9].

Identifying ophthalmic adverse events in patients on anti-HER2 therapy is critical for early detection and symptom management. Our case demonstrates a patient with transient ocular symptoms after treatment with pertuzumab-trastuzumab combination therapy and trastuzumab monotherapy.

## Case Presentation

This case report is presented in accordance with the 2013 CARE checklist (see online suppl. material; for all online suppl. material, see https://doi.org/10.1159/cro/abzag014) and highlights a woman in her 50s who presented in 2020 with a new diagnosis of stage IV breast cancer, specifically right breast biopsy-proven grade 3, hormone-receptor negative, HER2-positive, Ki-67 57% invasive ductal carcinoma. The patient has no known relevant comorbidities, including autoimmune conditions, diabetes, prior ophthalmologic history, or pertinent exposures to medication, environment, or travel. Per a staging computed tomography (CT) scan of her chest, abdomen, and pelvis (CAP), she was found to have multiple lung nodules, at least 2 indeterminate adrenal nodules, and an indeterminate liver nodule. Lung biopsy confirmed estrogen receptor-negative (0%), progesterone receptor-negative (0%), HER2-positive (3+ by immunohistochemistry, fluorescence in situ hybridization ratio 3.59, and copy number 8.8) metastatic carcinoma consistent with breast primary. She received first-line treatment [2] with 6 cycles of docetaxel, trastuzumab, and pertuzumab starting within a month of diagnosis, and then was transitioned to maintenance pertuzumab and trastuzumab (HP) 4 months later (year 0, see Fig. 1). The dosing schedule was docetaxel starting dose 75 mg/m^2^, escalated to 100 mg/m^2^; trastuzumab loading dose 8 mg/kg, followed by 6 mg/kg every 3 weeks; and pertuzumab loading dose 840 mg, followed by 420 mg every 3 weeks. No dose modifications or reductions occurred, but treatment was intermittently held as detailed below. Since late 2021, her staging CT CAP scans have shown no evidence of active disease.

**Fig. 1.**
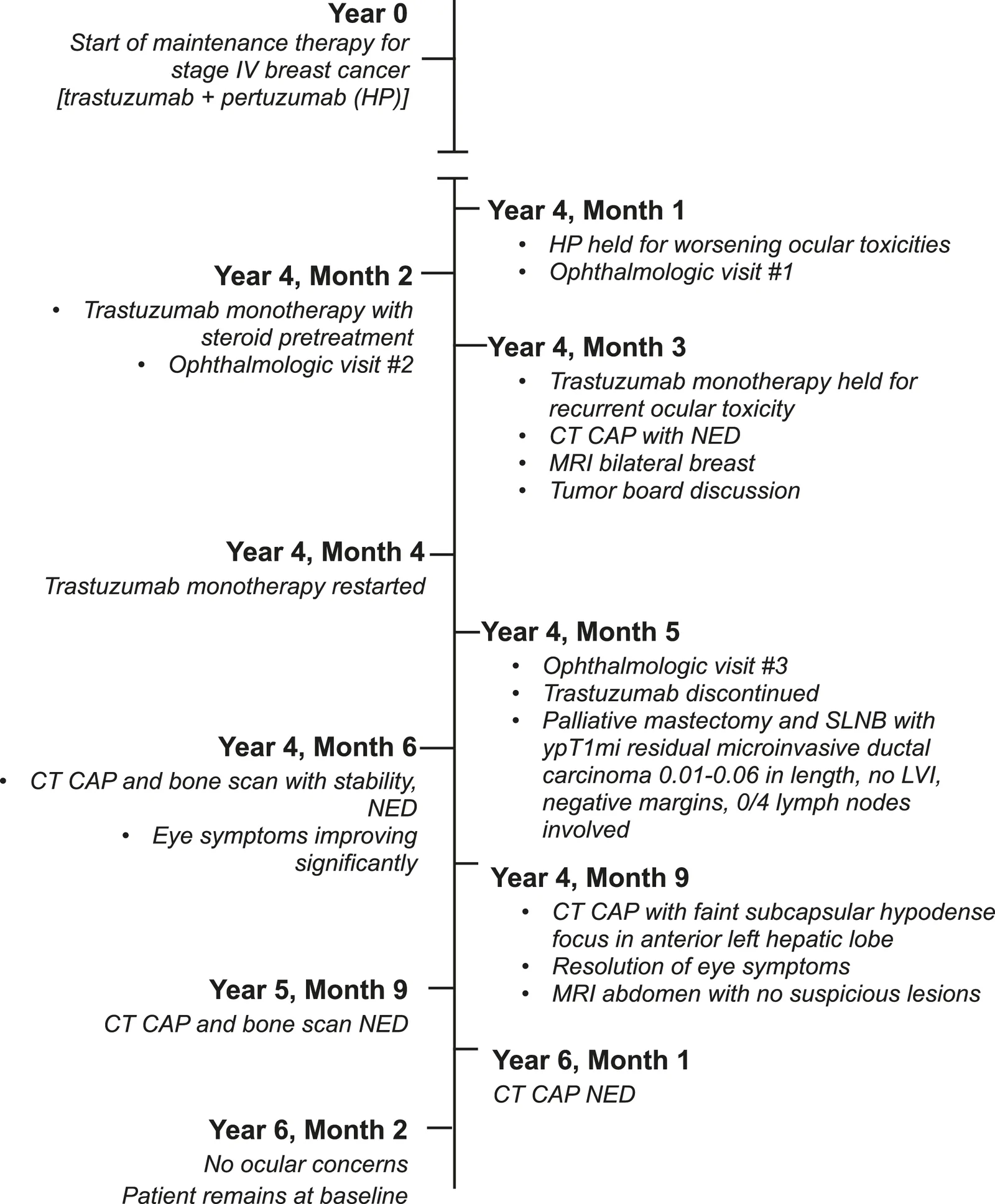
Treatment timeline.

However, she experienced several side effects with her treatment, including neuropathy, intermittent rash/hives with trastuzumab, diarrhea with HP, and diffuse joint pains. She noticed sporadic eye-related symptoms in late 2022, 2 years after starting maintenance therapy, that were initially attributed to eye dryness; however, with worsening symptoms in 2023, she sought medical attention. She noted new-onset bilateral conjunctivitis, dryness in the right eye, and blurry vision in the right eye. She reported nighttime eye pain and swelling, occasionally bilateral or unilateral, which would lead to difficulty with eyelid opening in the morning. Her maintenance treatment with HP was put on hold after discussing her symptoms with her oncologist (year 4, month 1). She was evaluated by an optometrist and diagnosed with dry eye syndrome of bilateral lacrimal glands and keratoconjunctivitis sicca, as evidenced by decreased visual acuity, decreased tear breakup time (<10 s), and superficial punctate keratitis with punctate epithelial erosions (Table 1, visit 1). She was prescribed prednisone eye drops with supportive care recommendations.

**Table 1.** Summary of significant ophthalmologic findings from outpatient appointments^a^

Optometry appointment date	Visit 1 (year 4, month 1)	Visit 2 (year 4, month 2)	Visit 3 (year 4, month 5)
Corrected visual acuity (OD, OS)	20/25, 20/100-1	20/40–1+1, 20/20	20/20, 20/20–2
Intraocular pressure (OD, OS)	14, 14	14, 13	11, 11
Tear breakup time (OU, s, normal: >10 s)	5	5	Not assessed
Conjunctival examination (OU)	1+ injection	Trace injection	White and quiet
Corneal examination (OU)	Mild diffuse SPK OS>OD, inferior PEEs	SPK improved, inferior PEEs	2+ PEE, reduced tear prism
Lens examination (OU)	No significant opacities	1–2+ NS OD>OS	1–2+ NS, with mild cortical changes in OS

a Table abbreviations: OD, right eye; OS, left eye; OU, both eyes; SPK, superficial punctate keratitis; PEE, punctate epithelial erosion; NS, nuclear sclerosis. Visual acuity definitition: Visual acuity: the top number denotes how far the patient is standing from the eye chart (20 feet). The bottom number denotes how far (in feet) a person with standard vision could read the same line on the eye chart; the greater it is above 20, the poorer the vision. A plus sign indicates the patient could read the main line as well as extra letters on the line below. A minus sign indicates the patient missed a few letters on the line.

After holding HP for a month, she was rechallenged with trastuzumab monotherapy with steroid pretreatment and continued to take prednisone drops for 8 days. During her optometry appointment in year 4, month 2, 8 days after trastuzumab rechallenge, aside from a new diagnosis of bilateral age-related nuclear cataracts, which was likely a contributing factor to her reduced vision, her ocular symptoms and examination findings improved off HP treatment (Table 1, visit 2). However, her symptoms later recurred, with a rechallenge of trastuzumab monotherapy after her second optometry visit. Treatment was thus held again in year 4, month 3, and she demonstrated improved examination findings at her follow-up appointment in year 4, month 5, 2 months after holding treatment (Table 1, visit 3).

In terms of notable imaging in year 4, month 3, her bilateral breast MRI showed overall improvement in her malignancy but noted a residual mass and non-mass enhancement in the right breast. The differential included malignancy or scar. Her CT CAP showed no evidence of metastatic disease in the chest, and no adenopathy in the abdomen or pelvis, and her bone scan showed no evidence of osteoblastic metastatic disease. The Multidisciplinary Tumor Board reviewed her case. Given the severity of her ocular symptoms, interference with quality of life, suspicious breast findings on imaging, and no evidence of distant disease per her staging scans obtained in year 4, month 3, the board recommended referral to surgical oncology for consideration of palliative mastectomy. She received 1 cycle of trastuzumab rechallenge with steroid pretreatment in year 4, month 4, before her surgery in year 4, month 5. She also stopped her steroid eye drops shortly before her surgery. She underwent an uncomplicated right mastectomy and sentinel lymph node biopsy with residual microinvasive disease (ypT1miypN0).

A month after her surgery, she reported significant improvement in ophthalmic symptoms, and her CT CAP and bone scan were stable, continuing to show no evidence of active disease. She and her medical oncologist discussed disease management options including close observation or a trial of a different anti-HER2 agent, as well as the risk of progression off therapy. Given the small, anticipated recurrence risk with all anti-HER2 agents based on Chen et al.’s [6] review of ocular adverse effects and the significant impact of her symptoms on her quality of life, the patient strongly preferred a nonsystemic therapy option. Additionally, her symptoms recurred with restarting trastuzumab despite steroid prophylaxis and close follow-up with optometry. The patient, thus, opted for close observation as opposed to alternative systemic therapies or continued steroid prophylaxis. In late 2024, she described her overall vision as different from before the onset of her ocular manifestations; however, she endorsed complete resolution of eye pain, dryness, swelling, and inability to open eyes, with continued improvement in vision. At the time of writing this report, about 2 years since discontinuing treatment, the patient’s staging scans continue to show no evidence of disease, and she denies recurrence of her symptoms. Written informed consent was obtained from the patient to publish this case report and any accompanying images.

## Discussion

This case demonstrates new-onset ocular symptoms during HP treatment that improved upon cessation of the medication and recurred with trastuzumab rechallenge. The temporal relationship of symptoms suggests that HP, specifically trastuzumab, was likely associated with the patient’s toxicities.

The likelihood that trastuzumab contributed to the patient’s symptoms can be characterized by the World Health Organization and Uppsala Monitoring Centre (WHO-UMC) system. Based on these criteria, the association would most strongly fall under the category of “probably/likely,” given the temporal relationship between symptoms and drug intake, the lower (though not definitive) likelihood of the toxicity being attributed to alternative causes, and the response to stopping the drug was clinically reasonable. Rechallenge is not required for this category, so the fact that our patient underwent a rechallenge further strengthens the association [10].

Ocular toxicities associated with pertuzumab alone are not well-documented. Since pertuzumab is always given in combination with trastuzumab in the clinical setting, it is difficult to isolate its effects, if any, on eye-related symptoms. Ma et al. [11] attempted to bridge this gap through their findings of blurred vision in healthy Chinese male participants after a single injection of pertuzumab. They noted significant limitations, however, due to a small sample size and the fact that pertuzumab targets HER2 in malignant breast cells of HER2-positive patients but will accumulate in the eyes of healthy participants, potentially leading to higher toxicity rates in healthy participants [11]. Furthermore, pertuzumab has a prolonged half-life, so residual drug exposure may have persisted during parts of the trastuzumab rechallenge. Therefore, although trastuzumab is the likely suspected toxicity-associated agent, pertuzumab or other contributing factors such as cataracts and age-related vision changes cannot be fully excluded.

When compared to the case reported by Saleh et al., both reports highlight ophthalmic damage that improved with holding the medication and recurred on rechallenge; however, the ophthalmologic diagnosis differed. Saleh et al. [8] noted a case of bilateral ischemic maculopathy and severe visual loss, whereas our patient experienced bilateral dry eye syndrome and keratoconjunctivitis sicca. Saleh et al.’s [8] patient had a visual acuity of 20/100 and 20/200 at symptom onset, while our patient had a visual acuity of 20/25 and 20/100-1 at symptom onset, in the right eye and left eye, respectively. This highlights that our patient had less severe vision impairment. Moreover, Salah et al.’s [8] patient developed symptoms after 3 months on the drug, while our patient developed symptoms 2 years after medication initiation.

In a case report by Peeters et al. [7], the patient developed corneal melting after 3 cycles of trastuzumab monotherapy, which improved with intense lubrication, topical antibiotics, and topical steroids. The patient was thus able to tolerate continued trastuzumab treatment with low-dose steroids and no progression of the corneal melting. In contrast, our patient’s symptoms persisted despite steroids, and she opted for surveillance in lieu of trialing a different treatment [7].

Another case report by Barmas-Alamdari et al. [9] outlines a patient with metastatic breast cancer undergoing treatment with trastuzumab who developed bilateral corneal ulceration and perforation of an ulcer in the left eye. The diagnoses and onset differ between this case and that of our patient. Reversibility also differs – this patient underwent therapeutic keratoplasty and ocular surface therapy to improve her symptoms, whereas our patient’s symptoms improved primarily with cessation of the drug and a possible beneficial contribution from steroids [9]. Overall, these case reports highlight variability with ocular diagnoses, timing of symptom onset, and reversibility of symptoms.

Generally, discontinuation of the causative agent and symptomatic systemic therapy leads to the resolution of ocular adverse events induced by anti-HER2 agents, as demonstrated in our patient. Severe cases of ocular complications or the need to continue anti-HER2 therapy as a life-saving treatment may present additional obstacles. Given the lack of data, optimal treatment has not yet been established, so ongoing research and close collaboration with eye specialists is needed to investigate treatment regimens for suspected toxicities.

A key characteristic of this case is the development of eye-related symptoms 2 years into this patient’s treatment course. This finding poses the question of whether ocular toxicities are associated with the long-term use of HP. Given advancements in treatments and early detection, patients with metastatic breast cancer are more readily diagnosed, live longer, and thus receive more cycles of maintenance HP, which may uncover new toxicities over the course of their treatment [12]. This highlights the importance of ongoing and thorough screening of patients on systemic therapy for new or worsening adverse events.

This case also emphasizes that medical oncologists may be underdiagnosing, and patients may be underreporting, the ophthalmologic side effects of HER2-targeted therapies. Medical oncologists do not routinely conduct eye examinations during appointments. Conversely, patients may dismiss certain ocular symptoms as irrelevant to their cancer diagnosis or not concerning enough to mention during their appointments. Therefore, medical oncologists should inquire about and encourage patients on chemotherapy to report any new, perplexing, or persistent ophthalmic symptoms regularly at follow-up appointments and consider early referral to an eye specialist for further evaluation of any persistent or severe symptoms. In the interim, oncologists should consider holding the medication, assess for any change in symptoms, and rechallenge when appropriate. Cessation and rechallenge of the medication may be helpful in determining the association of the drug with the toxicity, as evidenced by the WHO-UMC criteria.

A key limitation in our understanding of HP is the lack of knowledge of its long-term adverse events, particularly in patients receiving ongoing therapy for many years. The onset and timing of symptoms after receiving therapy is unclear. Further research is needed to evaluate the long-term adverse events of these medications and whether certain toxicities are dose-dependent or time-dependent, highlighting the importance of research and supportive care focused on long-term stage IV cancer survivors. Another limitation is the inability to fully rule out etiologies beyond trastuzumab, such as pertuzumab, cataracts, or aging as factors contributing to the patient’s symptoms. Moreover, this is the experience of a single patient; findings would be strengthened in conjunction with additional case reports and systematic reviews. The ophthalmologic aspect of our report is constrained in terms of limits of documentation from the patient’s outpatient optometrist, reported vision testing that was done, and limited guidance on optimal ophthalmologic evaluation, testing, and follow-up for patients receiving HER2-directed therapies. Current literature also suggests an inconsistent correlation between subjective symptoms and objective examination findings in dry eye syndrome [13]. This can be due to variability in diagnostic tools, differences in the interpretations of eye examinations, the subjectivity of reported symptoms, and the natural fluctuation of the disease process [13]. Further research may aid in developing the best ophthalmologic practices for patients on anti-HER2 therapies.

In conclusion, we report a case of ocular toxicity associated with trastuzumab and pertuzumab therapy in the setting of metastatic HER2-positive breast cancer. Medical oncologists should regularly and thoroughly screen for new symptoms in patients on anti-HER2 therapies, including ocular changes. If ocular manifestations resolve with ophthalmologic treatment, it may be appropriate to continue anti-HER2 therapy. If they persist despite treatment, such as in our patient, a discussion regarding a risk-benefit assessment and future breast cancer recurrence may be warranted when evaluating the need for continued treatment. Notable aspects of our case include the late onset of symptoms during the patient’s treatment course and the lack of formal guidelines for uniform ophthalmological evaluation. Future clinical trials involving anti-HER2-agents should monitor ophthalmic symptoms closely to better characterize associated adverse events, including their onset, potential for reversibility, and appropriate preventative and treatment options.

## Patient Perspective

When managing a chronic disease, I value shared decision-making, particularly in the setting of toxicities when there is limited data and uncertainty on how to proceed. Quality of life is a high priority for me, so I appreciate being an active part of the treatment team and collaborating on a personalized care plan. For example, I worked with my team to incorporate lifestyle changes into my care by improving my diet, home environment, stress levels, and overall wellness. The ability to individualize my treatment plan allows me to feel a better sense of patient autonomy. My experience has shown me the importance of advocacy on the part of the patient as well as the need for further research on chronic toxicities in the treatment of stage IV breast cancer.

## Acknowledgment

The authors would like to thank Angela Dahlberg, editor at The Ohio State University Comprehensive Cancer Center Division of Medical Oncology, for editing this manuscript.

## Statement of Ethics

Ethical approval is not required for this study in accordance with local or national guidelines. Written informed consent was obtained from the patient to publish the details of their medical case and any accompanying images.

## Conflict of Interest Statement

The authors have no conflicts of interest to declare.

## Funding Sources

This study was not supported by any sponsor or funder.

## Author Contributions

The first draft of the manuscript was written by Lahari Ramagiri. Kai C.C. Johnson, Margaret E. Gatti-Mays, and Ashley P. Davenport commented on previous versions of the manuscript. All authors, including Lahari Ramagiri, Kai C.C. Johnson, Margaret E. Gatti-Mays, and Ashley P. Davenport, read and approved the final manuscript.

## Data Availability

The data that support the findings of this study are not publicly available due to the fact that they contain information that could compromise the privacy of the patient, but they are available from Dr. Ashley P. Davenport upon reasonable request.

## References

[1] Callahan R , Hurvitz S. Human epidermal growth factor receptor-2-positive breast cancer: current management of early, advanced, and recurrent disease. Curr Opin Obstet Gynecol. 2011;23(1):37–43. 10.1097/gco.0b013e3283414e87 PMC4307801 21500375

[2] Giordano SH , Franzoi MAB, Temin S, Anders CK, Chandarlapaty S, Crews JR, . Systemic therapy for advanced human epidermal growth factor receptor 2-positive breast Cancer: ASCO Guideline update. J Clin Oncol. 2022;40(23):2612–35. 10.1200/JCO.22.00519 35640077

[3] Gion M , Trapani D, Cortés A, Valenza C, Lin N, Cortés J, . Systemic therapy for HER2-Positive metastatic breast cancer: moving into a new era. Am Soc Clin Oncol Educ Book. 2022;42:1–11. 10.1200/EDBK_351222 35671434

[4] Trastuzumab . LexiDrug [Internet]. UpToDate Inc. [cited 2024 October 12]. Available from: https://www.wolterskluwer.com/en/solutions/uptodate/pro/lexidrug

[5] Pertuzumab . LexiDrug [Internet]. UpToDate Inc. [cited 2024 October 12]. Available from: https://www.wolterskluwer.com/en/solutions/uptodate/pro/lexidruG

[6] Chen H , Yang G, Ma J. Ocular toxicity associated with anti-HER2 agents in breast cancer: a pharmacovigilance analysis using the FAERS database. Int J Cancer. 2024;154(9):1616–25. 10.1002/ijc.34848 38216995

[7] Peeters D , Vereecken M, Denys H, Roels D. A case of corneal melting in a patient with HER2-Positive breast cancer. Case Rep Ophthalmol. 2022;13(3):1036–41. 10.1159/000527236 PMC9808133 36605044

[8] Saleh M , Bourcier T, Noel G, Speeg-Schatz C, Gaucher D. Bilateral macular ischemia and severe visual loss following trastuzumab therapy. Acta Oncol. 2011;50(3):477–8. 10.3109/0284186X.2011.555781 21303229

[9] Barmas-Alamdari D , Chaudhary H, Baghdasaryan E, Dua P, Cheela I. Trastuzumab-induced early corneal melt in HER2-positive breast cancer: a case report and review. Am J Case Rep. 2024;25:e945488. 10.12659/AJCR.945488 PMC11537276 39478296

[10] World Health Organization [Internet] . The use of the WHO-UMC system for standardised case causality assessment. Available from: https://www.who.int/docs/default-source/medicines/pharmacovigilance/whocausality-assessment.pdf

[11] Ma J , Chen W, Hu Z, Huang J, Guo C, Zou C, . Rare ocular toxicity induced by pertuzumab/QL1209 in healthy Chinese subjects: case reports and whole-exome sequencing analysis. Invest New Drugs. 2022;40(4):861–7. 10.1007/s10637-022-01256-0 35596833

[12] Weide R , Feiten S, Waßmann C, Rendenbach B, Braun U, Burkhard O, . Metastatic breast cancer: prolonging life in routine oncology care. Cancers. 2024;16(7):1255. 10.3390/cancers16071255 PMC11011127 38610931

[13] Clayton JA . Dry eye. N Engl J Med. 2018;378(23):2212–23. 10.1056/NEJMra1407936 29874529

